# Enhanced depletion of MLL-fusion proteins in acute leukemia: potential for improved therapeutic outcomes

**DOI:** 10.1186/s40164-024-00556-w

**Published:** 2024-08-16

**Authors:** Noelia Che, Sandra Cantilena, Remi Looi-Somoye, Danesh Sundar, Kent Fung, Jasper de Boer, Owen Williams

**Affiliations:** 1 Cancer Section, Developmental Biology and Cancer Department, UCL Great Ormond Street Institute of Child Health, London, UK; 2 Department of Haematology, UCL Cancer Institute, London, UK

**Keywords:** MLL, KMT2A, Acute myeloid leukemia,, Acute lymphoblastic leukemia

## Abstract

Rearrangements of the *MLL* (*KMT2A*) locus are associated with aggressive leukaemia of both myeloid and lymphoid lineages, that present profound therapeutic challenges in pediatric and adult patient populations. *MLL*-fusion genes resulting from these rearrangements function as driving oncogenes and have been the focus of research aimed at understanding mechanisms underlying their leukemogenic activity and revealing novel therapeutic opportunities. Inspired by the paradigm of depleting the PML-RARA fusion protein in acute promyelocytic leukemia using all-trans retinoic acid and arsenic trioxide, we conducted a screen to identify FDA-approved drugs capable of depleting MLL-fusion protein expression in leukemia cells. Previously, we reported potent anti-leukemia effects of disulfiram (DSF), identified through this screen. In the present study, we demonstrate that another hit compound, niclosamide (NSM), is also able to deplete MLL-fusion proteins derived from a range of different *MLL*-fusion genes in both acute myeloid (AML) and acute lymphoid (ALL) leukemias. Loss of MLL-fusion protein appeared to result from inhibition of global protein translation by NSM. Importantly, combination of DSF with NSM enhanced MLL-fusion protein depletion. This led to more profound inhibition of downstream transcriptional leukemogenic programs regulated by MLL-fusion proteins and more effective killing of both *MLL*-rearranged AML and ALL cells. In contrast, DSF/NSM drug combination had little impact on normal hematopoietic progenitor cell differentiation. This study demonstrates that two FDA-approved drugs with excellent safety profiles can be combined to increase the efficacy of MLL-fusion protein depletion and elimination of *MLL*-rearranged leukaemia.

**Supplementary Information:**

The online version contains supplementary material available at 10.1186/s40164-024-00556-w.

## To the editor,

*MLL*(*KMT2A*)-rearranged leukaemias represent a significant clinical challenge, with infant *MLL*-rearranged acute leukemia having a particularly dim prognosis [1, 2]. Given that encoded MLL-fusion proteins are essential for leukemia survival and progression [3, 4], targeting them offers a promising therapeutic strategy. Indeed, significantly higher cancer response rates and longer progression-free survival were seen by inhibiting fusions versus non-fusions [5]. However, direct targeting of MLL-fusion proteins has been elusive.

We previously developed a screen to identify compounds that deplete MLL-fusion proteins in leukemia cells, leading to identification of disulfiram (DSF) as a candidate for repurposing in *MLL*-rearranged leukemia therapy. DSF disrupts MLL-fusion protein binding to DNA by specifically targeting the N-terminal CXXC domain, crucial for its DNA interaction, resulting in MLL-fusion protein depletion and consequent silencing of leukemia-promoting transcriptional pathways [6]. Among several promising compounds identified in this screen, Niclosamide (NSM) demonstrated the most significant depletion of MLL-AF9 (Fig. S1A) [6]. NSM showed broad efficacy, reducing expression of various MLL fusion proteins in both acute myeloid leukemia (AML) and acute lymphoblastic leukemia (ALL) cells (Fig. S1B-E). In all cases, reduced expression of the fusion protein was accompanied by depletion of the N-terminal MLL fragment expressed from the non-rearranged allele (Fig. S1B-E).

**Fig. 1:**
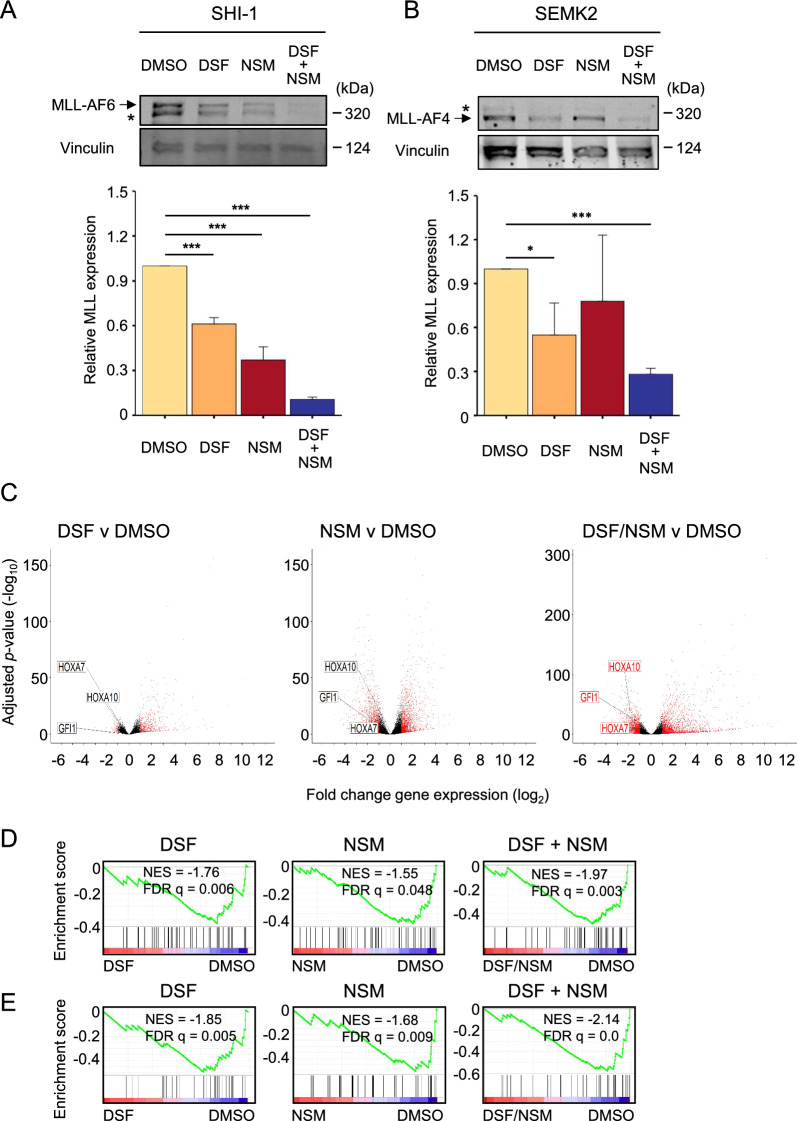
**A**, **B** Western blot examples (top panels) and quantification (lower panels) of the indicated MLL-fusion protein expression in **A** SHI-1 and **B** SEMK2 cells after 16 h exposure to DSF (0.15 µM DSF/1 µM Cu), NSM (A: 5 µM; B: 2.5 µM) or combined DSF + NSM. An (*) indicates the N-terminal wild type MLL band. Bars and error bars are means and SD of n = 3 independent experiments. Data are normalised to vinculin loading control and to DMSO treated control. *P < 0.05; ***P < 0.001, one sample *t*-test. **C** Volcano plots of RNA-seq analysis showing log_2_ fold gene expression changes versus adjusted *p*-value (−log_10_) in SHI-1 cells following 16 h treatment with DSF (0.3 µM DSF/1 µM Cu), NSM (5 µM) or combined DSF + NSM versus DMSO control, from n = 3 independent experiments. Expression changes greater than 1 (log_2_) are shown in red. **D**, **E** GSEA demonstrating negative enrichment of MLL-fusion target gene expression, defined as the overlap between MLL-AF9 target genes identified in THP-1 cells [9] and genes downregulated by inhibition of **D** MLL-AF9 [10] or **E** MLL-ENL and MLL-AF9 [11], in DSF, NSM and combined DSF + NSM induced gene expression changes

Exposure of *MLL*-rearranged cells to NSM led to inhibition of global translation, accompanied by increased eIF2α phosphorylation (Fig. S2A-E), in concordance with recent reports [7, 8]. NSM induced depletion of MLL-fusion proteins was reversible upon overexpression of a non-phosphorylatable eIF2α mutant, confirming the specific role of eIF2α phosphorylation in this pathway (Fig. S2E). These data are consistent with the effect of NSM on MLL-fusion protein expression being the result of a global inhibition of protein translation and thus not an effect that is specific to *MLL*-rearranged leukemia cells. NSM has pleiotropic effects on leukemia cells and has been shown to kill leukemia cells via different mechanisms [7]. However, given the susceptibility of MLL-fusion proteins to NSM, we explored whether NSM could enhance depletion of MLL-fusion proteins when combined with DSF. Indeed, combination of NSM and DSF, using concentrations that induced sub-optimal MLL-fusion protein depletion, enhanced the depletion of different MLL-fusion proteins in AML and ALL cells (Fig. 1A, B and Fig. S3A-B), in comparison to either drug alone. Each drug alone and in combination also induced depletion of the N-terminal MLL fragment expressed from the non-rearranged allele (Fig. S3C).

To determine whether this would result in more effective suppression of downstream transcriptional programs, RNA-sequencing was performed in MLL-AF6^+^ SHI-1 cells following 16-h exposure to NSM and DSF alone and in combination (Fig. 1C). Drug combination resulted in an increased number of significant gene expression changes, among which enhanced suppression of MLL-fusion target genes was evident, for example *HOXA7*, *HOXA10* and *GFI1* (Fig. 1C and Fig. S3D). Importantly, *KMT2A* expression was not decreased by any of the drug treatments (Fig. S3E). GSEA analysis of these global gene expression changes was then performed, using MLL-fusion target gene sets (Supplementary Table S1). The overlap between MLL-AF9 target genes identified in THP-1 cells [9] and genes downregulated by inhibition of MLL-AF9 [10] or MLL-ENL and MLL-AF9 [11] in mouse leukemia cells were used. Both genesets were negatively enriched in SHI-1 cells treated with NSM or DSF, combination treatment with NSM and DSF resulting in more significant negative enrichment (Fig. 1D, E). Our study demonstrates that the combination of NSM and DSF significantly suppresses key transcriptional pathways driven by MLL-fusion proteins, suggesting that such combination therapies could redefine treatment paradigms in leukemia characterized by *MLL* rearrangements.

**Fig. 2:**
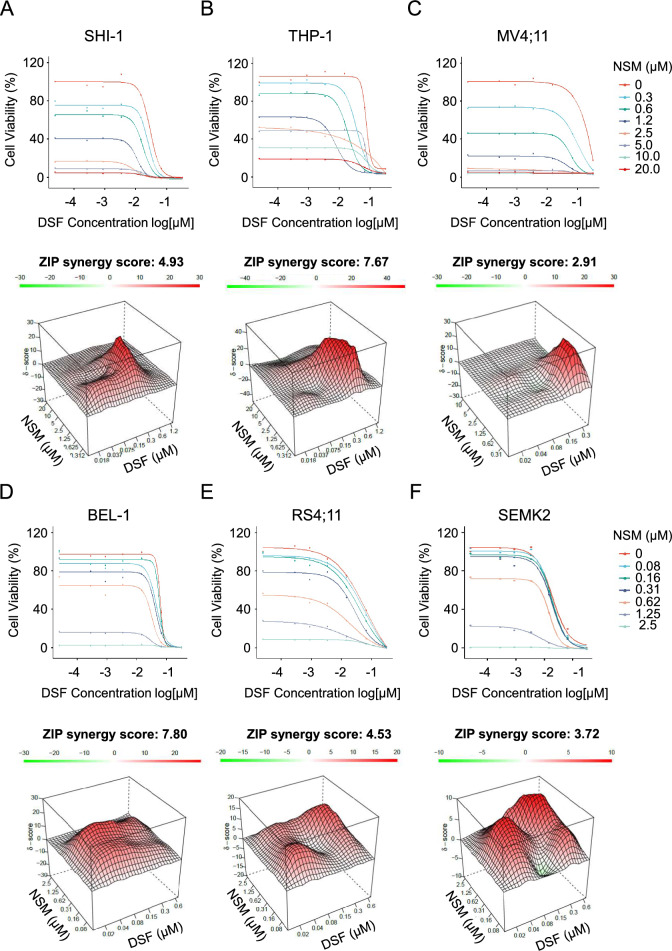
Viability (top panels) of **A** SHI-1, **B** THP-1, **C** MV4;11, **D** BEL-1, **E** RS4;11 and **F** SEMK2 cells following 72 h treatment with indicated concentrations of DSF (with 1 µM Cu) and NSM. Data are normalised to DMSO-treated cells. Graphs points are means of n = 3 independent experiments. 3D synergy maps and ZIP synergy scores (lower panels) of data calculated with SynergyFinder (version 2.0)

Next, we assessed whether the synergistic effects of NSM and DSF translate into enhanced anti-leukemic activity. Exposure of *MLL*-rearranged AML and ALL cells to NSM and DSF led to an additive loss in cell viability in all cell lines (Fig. 2). This was consistent with an increase in cell death resulting from exposure to the drug combination (Fig. S4). In contrast, the NSM/DSF combination had no significant effect on colony formation by normal CD34^+^ cord blood cells (Fig. S5), although the long-term consequences of combination drug exposure would have to be examined in future clinical trials.

In conclusion, our study demonstrates that the antihelminthic drug NSM enhances the activity of DSF in depleting MLL-fusion proteins, suppressing downstream transcriptional pathways and eradicating *MLL*-rearranged leukaemia cells [6]. Both NSM and DSF are clinically relevant, each with an excellent safety profile, supporting their potential for rapid clinical translation. A limitation of our study is that it is based on in vitro experiments only, given the difficulty in using DSF in mouse models in vivo [6]. However, DSF is under investigation in several different types of cancers, including metastatic breast cancer (NCT03323346), gastric cancer (NCT05667415) and refractory sarcomas (NCT05210374), and NSM is currently in clinical trial for relapsed and refractory paediatric AML (NCT05188170). Our findings position NSM and DSF as promising agents for the repurposing in the treatment of *MLL*-rearranged leukemia. Future clinical trials are warranted to comprehensively assess their efficacy and safety in patient populations.

### Supplementary Information

Additional file 1.

## Data Availability

The sequencing data discussed in this publication have been deposited in the National Center for Biotechnology Information Gene Expression Omnibus and are accessible through GEO Series accession number GSE262673 and are available at the following URL: https://www.ncbi.nlm.nih.gov/geo/query/acc.cgi?acc = GSE262673.

## Abbreviations

FDA: U.S. food and drug administration
DSF: Disulfiram
NSM: Niclosamide
AML: Acute myeloid leukemia
ALL: Acute lymphoblastic leukemia
