# Supplementary material for: Enhanced depletion of MLL-fusion proteins in acute leukemia: potential for improved therapeutic outcomes
Source: Exp Hematol Oncol. 2024 Aug 16;13:84. doi: 10.1186/s40164-024-00556-w (PMC11328480; doi:10.1186/s40164-024-00556-w)
Supplement: Supplementary file 1 — Additional file 1. [file 40164_2024_556_MOESM1_ESM.pdf]

Supplementary Materials and Methods

Cell lines and cell culture

Human leukaemic cell lines SEMK2, BEL-1, RS4;11, KOPN8, SHI-1, MV4-11 were obtained from the German Collection of Microorganisms and Cell Cultures (DSMZ). The THP-1 cell line was obtained from the American Type Culture Collection (ATCC, TIB-202, www.atcc.org). The identity of human leukaemic cell lines was confirmed by STR profiling. All human leukaemic cell lines were routinely cultured in Roswell Park Memorial Institute (RPMI) medium (Sigma-Aldrich), supplemented with 10% or 20% heat-inactivated FBS, penicillin-streptomycin (100µg/ml) and L-glutamine (2mM) (all Sigma-Aldrich) with the exception of SHI-1 cells which were cultured in Iscove's Modified Dulbecco's Medium supplemented with 20% heat-inactivated FBS, penicillin-streptomycin (100µg/ml) and L-glutamine (2mM) (all Sigma-Aldrich) in a humidified chamber with 5% CO_2 and 95% air at 37C. Each cell line was sub-cultured every 3-4 days and plated according to supplier guidelines. Cryopreserved human cord blood $CD34^+$ cells were from pooled donors (ZenBio Inc, NC, USA). Colony formation by $CD34^+$ cord blood cells was performed by plating cells in HSC005 (Bio Techne) methylcellulose medium and analysing colony number and morphology 14 days later. 293FT (ThermoFisher Scientific, Hemel Hempstead, UK) cells were maintained in DMEM with 10% heat-inactivated FBS, L-glutamine, penicillin/streptomycin and 500µg/ml G418.

Flow cytometry analysis

Apoptosis was determined by staining cells using the Annexin V Apoptosis Detection Kit APC (eBioscience) in combination with Propidium Iodide (PI). Both analyses were performed on a LSRII analyser (BD Bioscience).

Viability assay

Cell viability was evaluated with the CellTiter 96® AQueous One Solution Cell Proliferation Assay (Promega). ZIP synergy score was calculated using SynergyFinder (version 2.0) online software.[1]

Western Blot analysis

Cell lysates were prepared using a sample reducing buffer containing DTT (200mM), sodium dodecyl sulphate (SDS) (2%), Glycerol (10% v/v), Bromophenol Blue (0.02% v/v), Tris-HCl pH6.8 (125 mM). Samples were pelleted via centrifugation and equal amounts of protein were resolved in a 7% Bis-Tris polyacrylamide gel. Proteins were transferred to nitrocellulose membranes (LI-COR Biosystem). Membranes were blocked using 5% dry non-fat milk in 0.5% Tween 20 in PBS and then probed using the following antibodies: MLL^N/HRX (clone N4.4, Millipore), vinculin (clone EPR8185, Abcam), anti-EIF2S1 (ab13762, Abcam), anti-phospho-EIF2S1 (ab131505, Abcam) and α-Tubulin (YL1/2, catalogue number sc-53029, Santa Cruz Biotechnology). Membranes were imaged using Odyssey CLX imaging system (LI-COR Biosystem). Full unmodified western blot images are shown in Fig. S6-8.

Lentivirus vector constructs

The wild-type (eIF2α^{WT}) and mutated eIF2α (eIF2α^{S51A}) overexpression constructs were a gift from David Ron (eIF2a 1 and 2, Addgene plasmids #21807 and #21808), and were cloned into the pCSGW-PIG lentivirus expression construct.[2]

RNA isolation and qRT-PCR

Total RNA was isolated using the RNAeasy Mini Kit (Qiagen) and RNA concentration was measured using a NanoDrop ND-1000 (Labtech International). RNA (1 µg) was converted in cDNA using the High Capacity RNA-to-cDNA kit (Thermo Fisher) according to manufacturer's instructions. Quantitative RT-PCR was performed and analyzed using the StepOnePlus™ Real-Time PCR system (Life Technologies). Relative expression was calculated using the ΔΔCt method and the *RPL19* gene was used as the reference gene. Primer/probe sets were from Applied Biosystems, Life Technologies.

Library preparation, RNA sequencing and data analysis

RNA was purified from control, NSM, DSF or combination (NSM/DSF) treated SHI-1 cells from three independent experiments each and submitted to UCL Genomics for RNA-sequencing. Total cellular RNA was extracted using the RNeasy Plus Mini Kit (Qiagen, Manchester, UK) according to manufacturer's instructions. 100 ng of RNA per sample were analysed using Bioanalyser 2100 (Agilent Technologies, Santa Clara, CA) to verify RNA integrity prior to amplification. Samples were processed using the Illumina TruSeq RNA sample prep kit Version2 (p/n RS-122-2001) according to manufacturer's instructions (Illumina, Cambridge, UK). Briefly, mRNA was selected using paramagnetic dT beads and fragmented by metal hydrolysis to approximately 150 bp lengths. Random primed cDNA was then generated and adapters compatible

with Illumina sequencing were ligated before being enriched by 12 cycles of PCR. Libraries were quantified, normalised, and pooled before sequencing on an Illumina NextSeq 500, generating approximately 20 million 43 bp read pairs per sample. Fastq was then demultiplexed and generated using Illumina bcl2fastq v2.19 before pre-processing (trimmomatic) to remove adapter read-through and poor-quality sequences. Alignment and quantification were performed with STAR[3] and Salmon[4] using nf-core/rnaseq pipeline version 3.12[5]. NCBI GRCh38 on Illumina iGenomes collection was used as reference genome. Differential gene expression analysis was performed using R package DESeq2 (R version 4.2.1, DESeq2 version 1.36.0)[6,7].

Gene set enrichment analysis (GSEA)

Gene expression data were analyzed for enrichment using GSEA software (Broad Institute-version 4.3.3). GSEA ranked a list of normalized RNAseq data according to the expression difference (signal-to-noise ratio) and calculates an enrichment score (ES) by walking down this list and increasing a running sum statistic when it encounters a member within the gene set definition. Conversely, this statistic decreases when encountering a gene not in the gene set. The maximum deviation from zero constitutes the ES and corresponds to a weighted Kolmogorov–Smirnov-like statistic. Once all gene sets have been evaluated, GSEA adjusts the estimated significance level to account for multiple hypothesis testing and adjusts for the respective sizes of the gene sets, ultimately generating a NES. Use of the NES facilitates comparison across gene sets.

Protein Synthesis Assay

RS4;11 and THP1 cells were labelled with O-Propargyl-puromycin (OPP) probe (Cayman Chemical #601100) and treated with NSM at 37˚C for 4 hours. Cells were then fixed and washed according to manufacturer's protocol. Cells were subsequently stained with 5 FAM-Azide for 30 minutes at room temperature in the dark followed by flow cytometry analysis.

Statistics

Statistical significance was determined using Prism (GraphPad) software. Statistical analysis of means was performed using the one sample t test or unpaired Student's t test, two-tailed *P* values < 0.05 being considered statistically significant. Variance was similar between groups. Statistical analysis of survival curves was performed using the log-rank test.

Data and materials availability

The RNAseq data discussed in this publication have been deposited in NCBI's Gene Expression Omnibus and are accessible through GEO Series accession number GSE262673.

References

1. Ianevski A, Giri AK, Aittokallio T. SynergyFinder 2.0: visual analytics of multi-drug combination synergies. Nucleic Acids Res. 2020;48(W1):W488-W493.
2. Walf-Vorderwulbecke V, Pearce K, Brooks T, Hubank M, van den Heuvel-Eibrink MM, Zwaan CM, et al. Targeting acute myeloid leukemia by drug-induced c-MYB degradation. Leukemia. 2018;32:882-889.

3. Dobin A, Davis CA, Schlesinger F, Drenkow J, Zaleski C, Jha S, Batut P, Chaisson M, Gingeras TR. STAR: ultrafast universal RNA-seq aligner. Bioinformatics. 2013 Jan 1;29(1):15-21. doi: 10.1093/bioinformatics/bts635. Epub 2012 Oct 25.
4. Patro, R., Duggal, G., Love, M. I., Irizarry, R. A., & Kingsford, C. (2017). Salmon provides fast and bias-aware quantification of transcript expression. Nature Methods, 14, 417–419. DOI: 10.1038/nmeth.4197
5. Philip Ewels, Alexander Peltzer, Sven Fillinger, Harshil Patel, Johannes Alneberg, Andreas Wilm, Maxime Ulysse Garcia, Paolo Di Tommaso & Sven Nahnsen. The nf-core framework for community-curated bioinformatics pipelines. Nat Biotechnol. 2020 Feb 13. doi: 10.1038/s41587-020-0439-x.
6. R Core Team (2022). R: A language and environment for statistical computing. R Foundation for Statistical Computing, Vienna, Austria. Retrieved from https://www.R-project.org/
7. Love, M.I., Huber, W., Anders, S. (2014). Moderated estimation of fold change and dispersion for RNA-seq data with DESeq2. Genome Biology, 15, 550. DOI: 10.1186/s13059-014-0550-8.

Supplementary Figure Legends

Fig. S1 A The MLL-fusion depletion screen performed using the Prestwick clinical compound library. Cells were treated with 10μM of each compound for 6h and MLL-fusion levels were measured using a dual-luciferase assay. Normalized Fluc/Rluc ratios are plotted. 13 compounds showed a reduction larger than 33% in normalized Fluc activity. DSF and NSM are indicated with the green circles. **B**-**E** Western blot examples (top panels) and quantification (lower panels) of the indicated MLL-fusion protein expression in **B** SHI-1, **C** SEMK2, **D** BEL-1 and **E** KOPN8 cells after 16h exposure to 5μM NSM. An (*) indicates the N-terminal wild type MLL band. Bars and error bars are means and SD of n=3 (SHI-1, SEMK2, BEL-1) and n=4 (KOPN8) independent experiments. Data are normalised to vinculin loading control and to DMSO treated control. $^{**}P < 0.01$; $^{***}P < 0.001$, one sample *t*-test.

Fig. S2 A,**B** Examples of flow cytometric analysis (left panels) and quantification (right panels) of OPP fluorescence in **A** RS4;11 and **B** THP-1 cells exposed to 4h 5μM NSM. Bars and error bars are means and SD of n=3 independent experiments. Data are to DMSO treated control. $^{***}P < 0.001$, one sample *t*-test. **C**,**D** Western blot example (left panels) and quantification (right panels) of phospho-eIF2α and total eIF2α protein expression in **C** RS4;11 and **D** THP-1 cells exposed to 4h 5μM NSM. Bars and error bars are means and SD of n=4 independent experiments. Data are normalised to total eIF2α protein and to DMSO treated control. $^{*}P < 0.05$; $^{***}P < 0.001$, one sample *t*-test. **E** OPP fluorescence in RS4;11 cells exposed to 4h 5μM NSM or 25μg/ml cycloheximide. **F** Western blot example (left panel) of MLL-AF9, phospho-eIF2α and total eIF2α protein expression in THP-1 cells, transduced with empty vector, wild-type

eIF2α or mutant eIF2α (eIF2α^{S51A}), after 16h exposure to 5μM NSM or DMSO control. An (*) indicates the N-terminal wild type MLL band. Quantification of MLL-AF9 expression (right panel) normalised to vinculin loading control and to DMSO treated control. Bars and error bars are means and SD of n=3 independent experiments. *P < 0.05; **P < 0.01; n.s. not significant, one sample *t*-test. Data are normalised to vinculin loading control and to DMSO treated control.

Fig. S3 A,**B** Western blot examples (top panels) and quantification (lower panels) of the indicated MLL-fusion protein expression in **A** RS4;11 and **B** BEL-1 cells after 16h exposure to DSF (0.15μM DSF/1μM Cu), 2.5μM NSM or combined DSF + NSM. An (*) indicates the N-terminal wild type MLL band. Bars and error bars are means and SD of n=3 independent experiments. Data are normalised to vinculin loading control and to DMSO treated control. *P < 0.05; **P < 0.01, ***P < 0.001, one sample *t*-test. **C** Quantification of changes in MLL-AF6 fusion protein compared to N-terminal MLL fragment expression from data in Fig. 1A, in SHI-1 cells. **D** Example of qRT-PCR analysis of *HOXA10* gene expression in SHI-1 cells 16h after exposure to DSF (0.15μM DSF/1μM Cu), 5μM NSM or combined DSF + NSM. Bars and error bars are means and SD of n=3 independent experiments. Gene expression data are normalised to DMSO-treated cells. *P < 0.05; **P < 0.01; ****P < 0.0001, one sample *t* test. **E** Log2 fold change in expression of *KMT2A* from RNA-sequencing, in DSF (adjusted P = 0.004), NSM (adjusted P = 2.1e-20) and combined DSF + NSM (adjusted P = 6.2e-07) treated groups in comparison to DMSO treated cells.

Fig. S4 Examples (left panels) and quantifications (right panels) of flow cytometric analysis of PI and Annexin V in **A** SEMK2, **B** RS4;11 and **C** BEL-1 cells treated for

48h with DSF (A,C: 0.075μM DSF/1μM Cu; B: 0.15μM DSF/1μM Cu), NSM (A: 1.25μM; B: 2.5μM; C: 0.625μM) or combined DSF + NSM. Bars and error bars are means and SD of n=3 independent experiments. $^{*}P < 0.05$; $^{***}P < 0.001$, unpaired Student's *t* test between drug-treated and DMSO-treated cells.

Fig. S5 Quantification of colony formation by $CD34^{+}$ cord blood cells in the presence of DSF (0.15μM DSF/1μM Cu), 5μM NSM or combined DSF + NSM. Bars and error bars are means and SD of n=2 independent experiments.

Fig. S6 Full unmodified western blots for Fig. 1 A and B and Fig. S3 A and B.

Fig. S7 Full unmodified western blots for Fig. S2 C and D.

Fig. S8 Full unmodified western blots for Fig. S2 F.

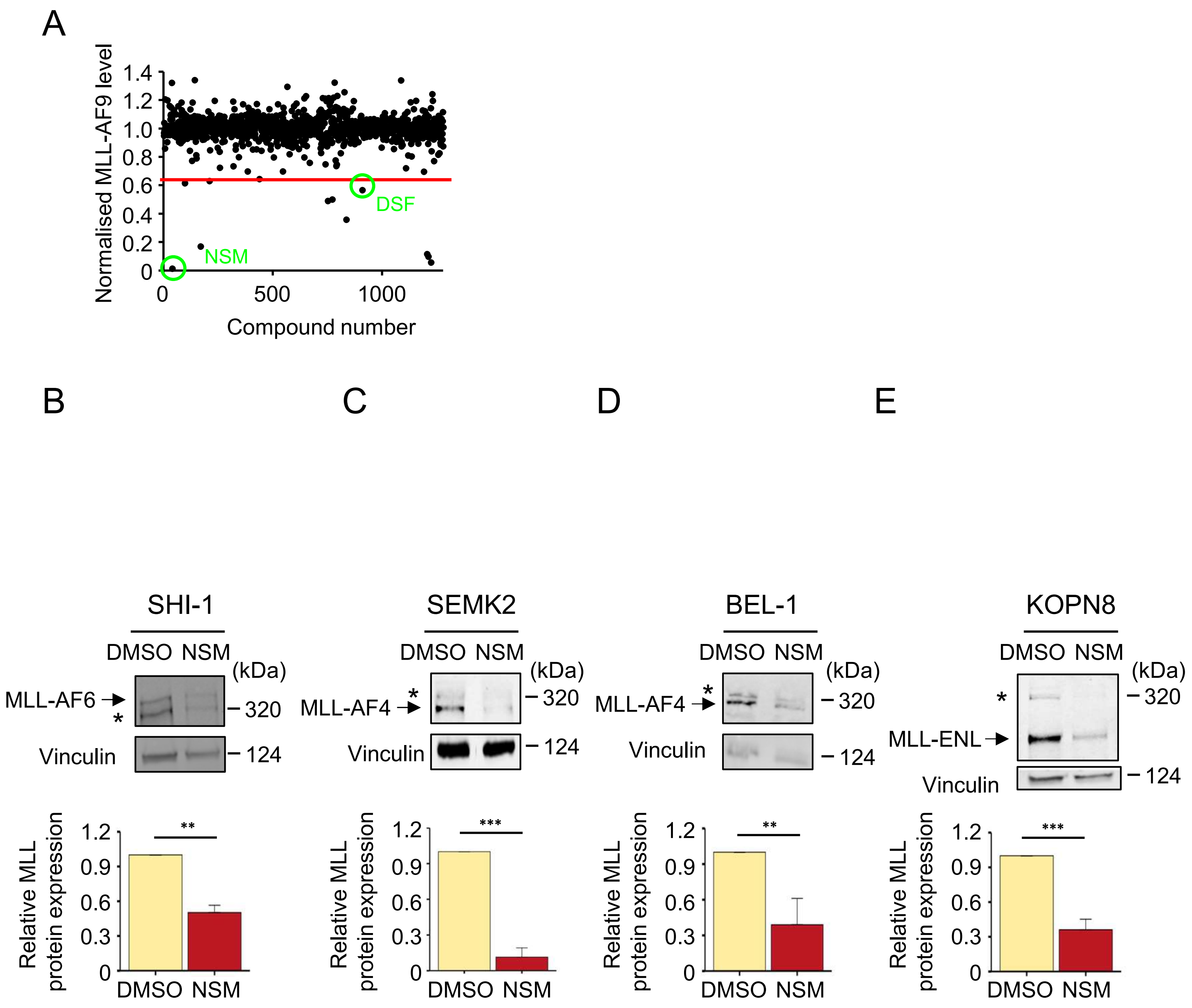
A
Normalised MLL-AF9 level
1.4
1.2
1.0
0.8
0.6
0.4
0.2
0
DSF
NSM
0
500
1000
Compound number
B
C
D
E
SHI-1
SEMK2
BEL-1
KOPN8
DMSO NSM
(kDa)
MLL-AF6
MLL-AF4
MLL-ENL
*
320
Vinculin
124
Relative MLL protein expression
1.2
0.9
0.6
0.3
0
**
***
DMSO NSM

Figure S1

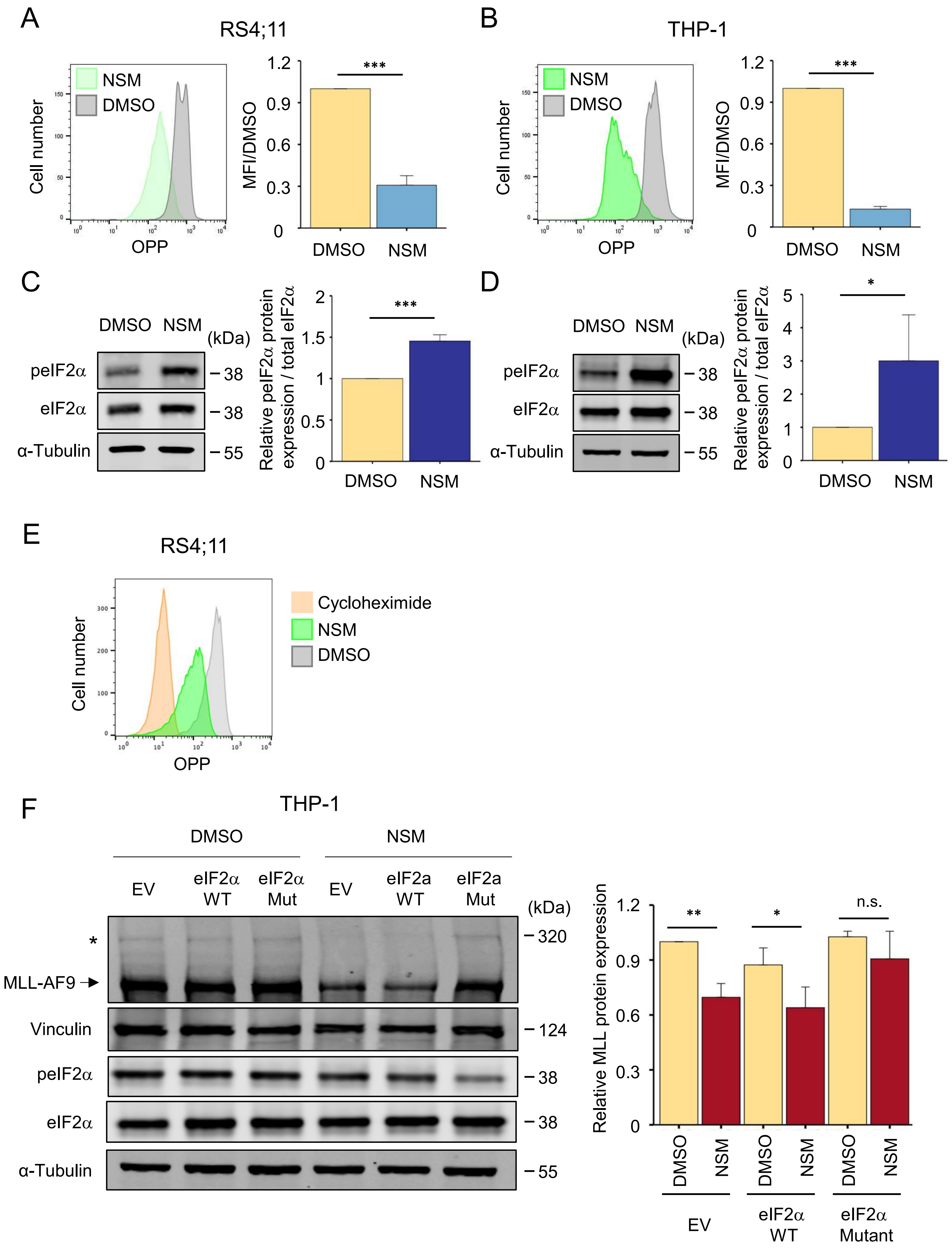
A
RS4;11
NSM
DMSO
Cell number
OPP
MFI/DMSO
***
DMSO
NSM
B
THP-1
NSM
DMSO
Cell number
OPP
MFI/DMSO
***
DMSO
NSM
C
DMSO NSM
(kDa)
peIF2α
eIF2α
α-Tubulin
38
38
55
Relative peIF2α protein expression / total eIF2α
***
DMSO
NSM
D
DMSO NSM
(kDa)
peIF2α
eIF2α
α-Tubulin
38
38
55
Relative peIF2α protein expression / total eIF2α
*
DMSO
NSM
E
RS4;11
Cycloheximide
NSM
DMSO
Cell number
OPP
F
THP-1
DMSO
NSM
EV
eIF2α WT
eIF2α Mut
EV
eIF2a WT
eIF2a Mut
(kDa)
*
MLL-AF9
Vinculin
peIF2α
eIF2α
α-Tubulin
320
124
38
38
55
Relative MLL protein expression
**
*
n.s.
DMSO
NSM
DMSO
NSM
DMSO
NSM
EV
eIF2α WT
eIF2α Mutant

Figure S2

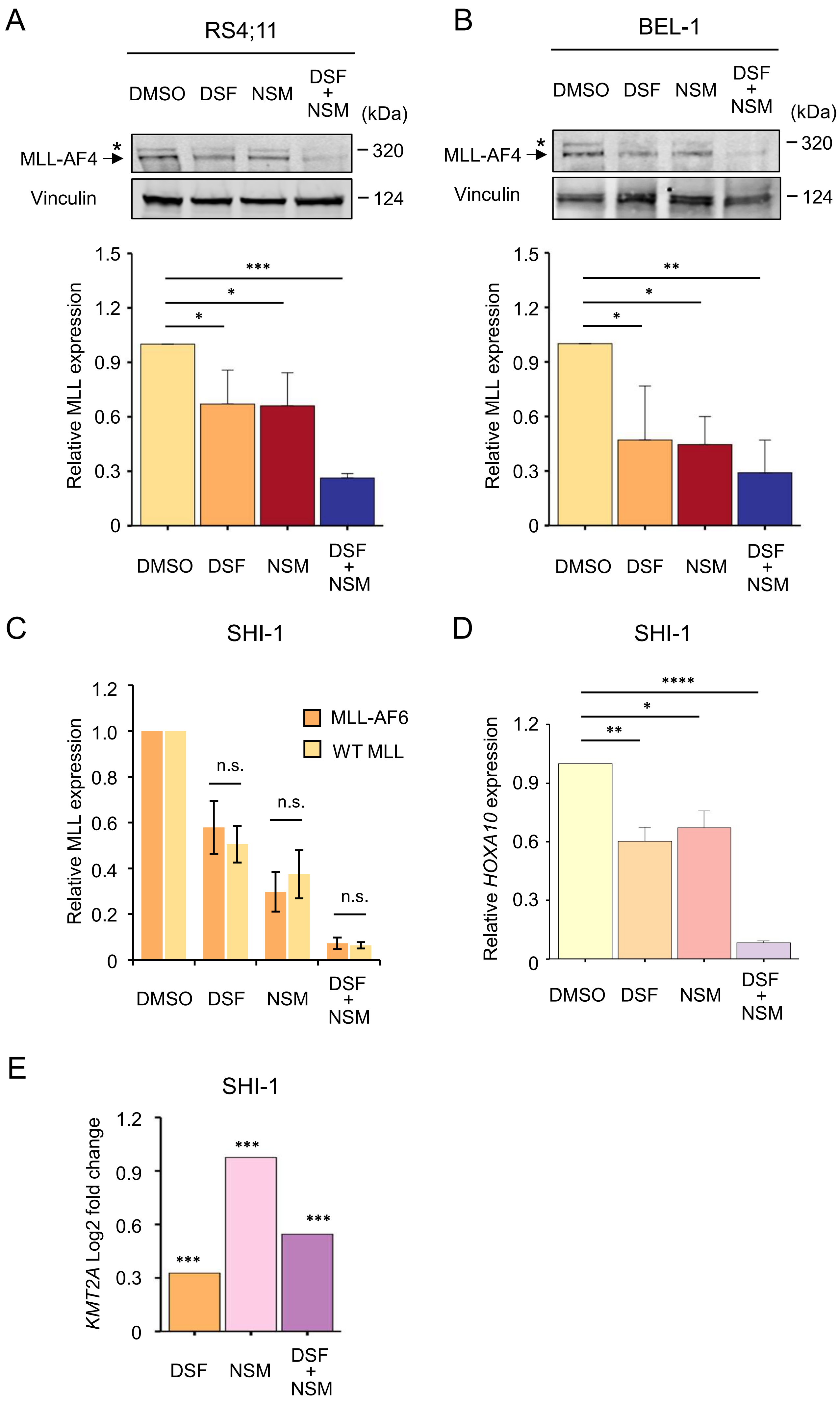
A
RS4;11
DMSO DSF NSM DSF + NSM (kDa)
MLL-AF4
*
Vinculin
320
124
Relative MLL expression
1.5
1.2
0.9
0.6
0.3
0
***
*
*
DMSO DSF NSM DSF + NSM
B
BEL-1
DMSO DSF NSM DSF + NSM (kDa)
MLL-AF4
*
Vinculin
320
124
Relative MLL expression
1.5
1.2
0.9
0.6
0.3
0
**
*
*
DMSO DSF NSM DSF + NSM
C
SHI-1
Relative MLL expression
1.2
1.0
0.8
0.6
0.4
0.2
0
MLL-AF6
WT MLL
n.s.
n.s.
n.s.
DMSO DSF NSM DSF + NSM
D
SHI-1
Relative HOXA10 expression
1.2
0.9
0.6
0.3
0
****
*
**
DMSO DSF NSM DSF + NSM
E
SHI-1
KMT2A Log2 fold change
1.2
0.9
0.6
0.3
0
***
***
***
DSF NSM DSF + NSM

Figure S3

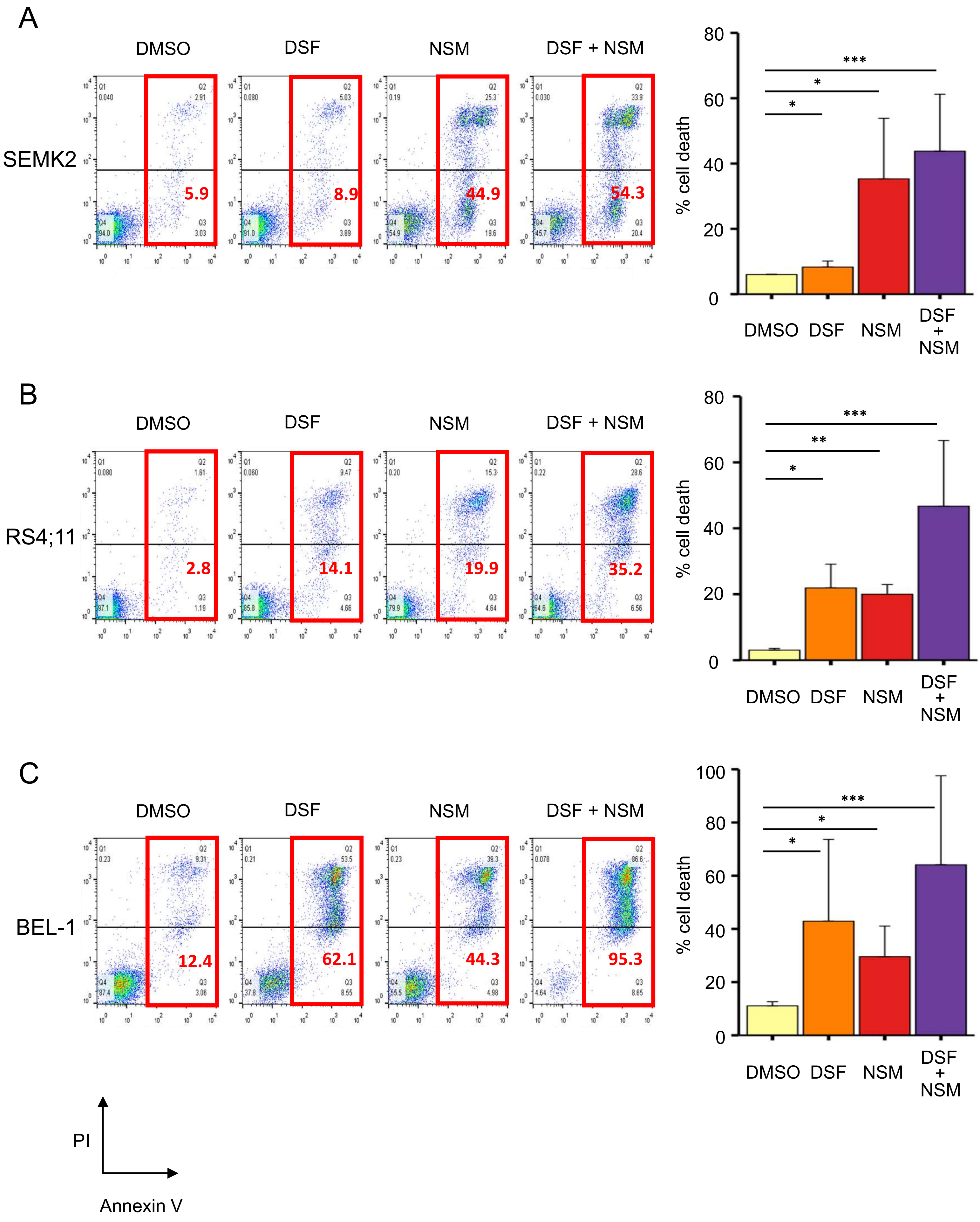
A
B
C
DMSO
DSF
NSM
DSF + NSM
SEMK2
RS4;11
BEL-1
5.9
8.9
44.9
54.3
2.8
14.1
19.9
35.2
12.4
62.1
44.3
95.3
% cell death
DSF
+
NSM
PI
Annexin V

Figure S4

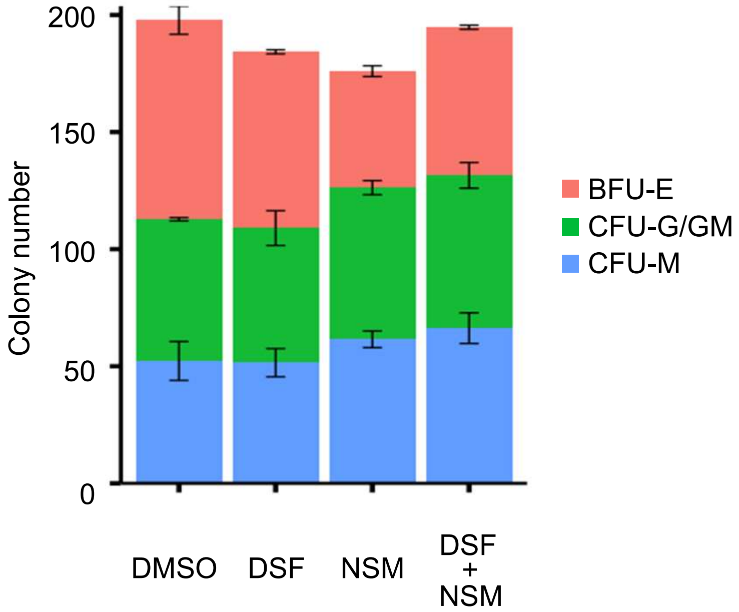
200
150
100
50
0
Colony number
DMSO
DSF
NSM
DSF
+
NSM
BFU-E
CFU-G/GM
CFU-M

Figure S5

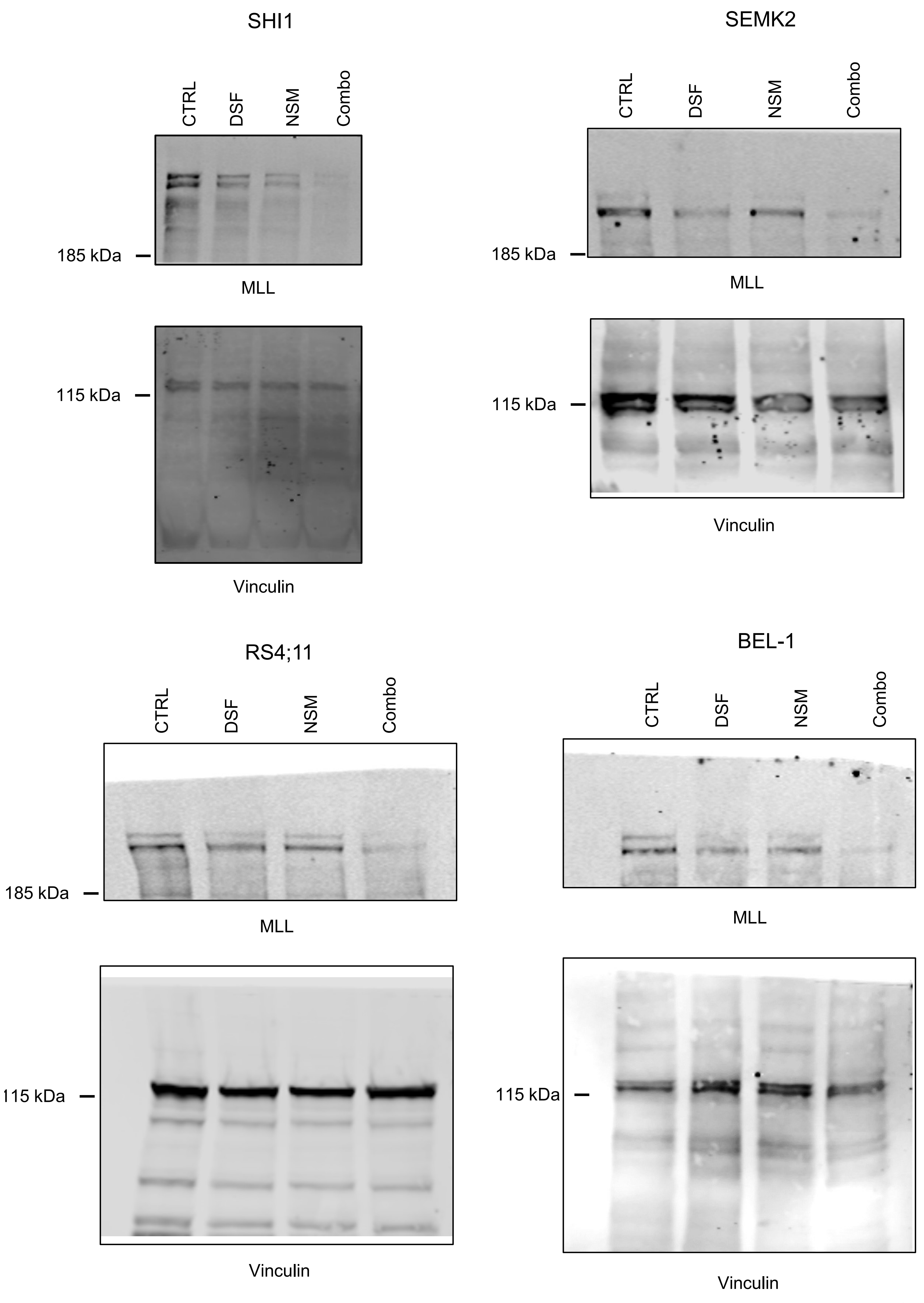
SHI1
CTRL
DSF
NSM
Combo
185 kDa
MLL
115 kDa
Vinculin
SEMK2
CTRL
DSF
NSM
Combo
185 kDa
MLL
115 kDa
Vinculin
RS4;11
CTRL
DSF
NSM
Combo
185 kDa
MLL
115 kDa
Vinculin
BEL-1
CTRL
DSF
NSM
Combo
MLL
115 kDa
Vinculin

Figure S6

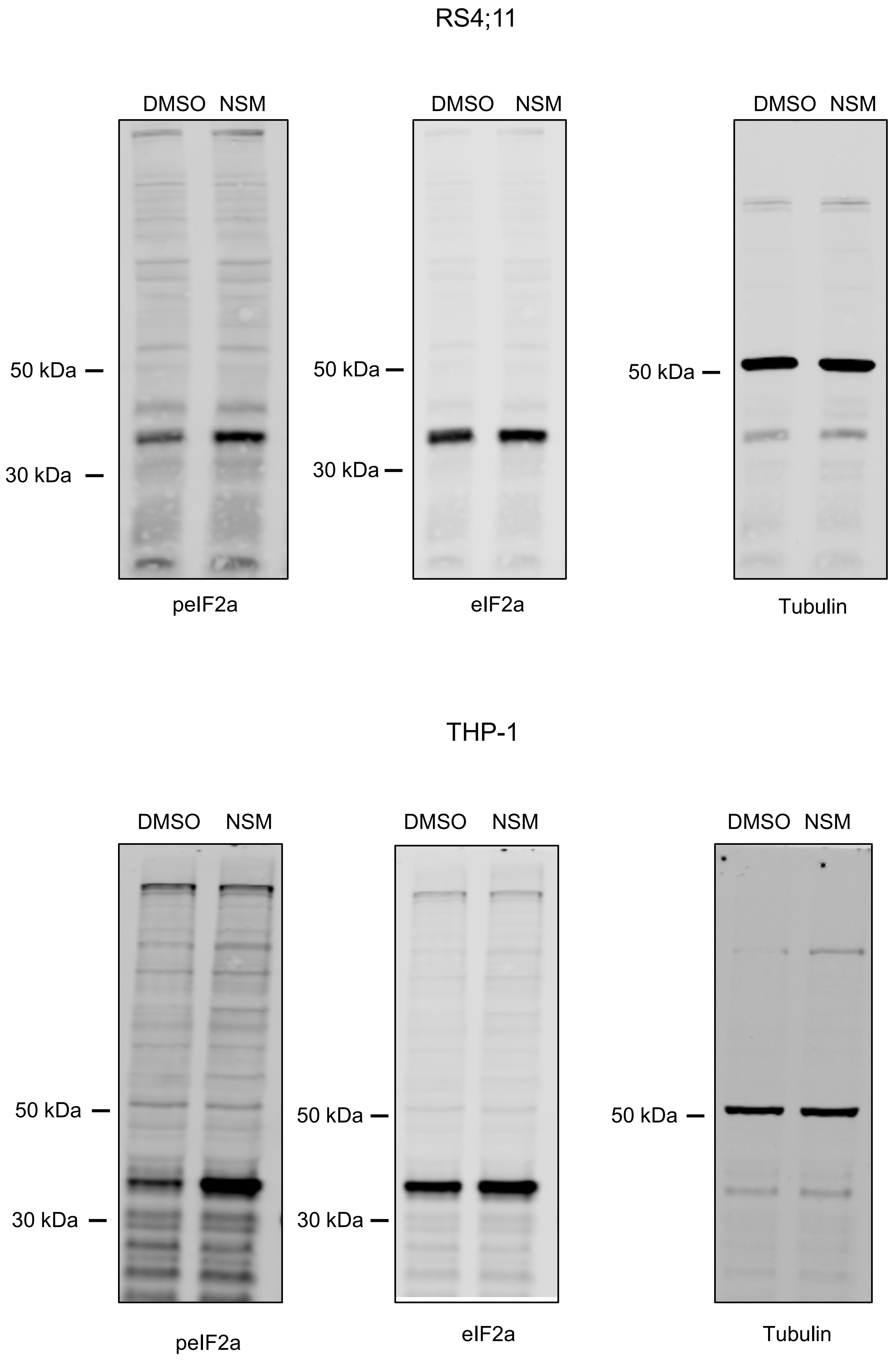
RS4;11
DMSO NSM
DMSO NSM
DMSO NSM
50 kDa
30 kDa
50 kDa
30 kDa
50 kDa
peIF2a
eIF2a
Tubulin
THP-1
DMSO NSM
DMSO NSM
DMSO NSM
50 kDa
30 kDa
50 kDa
30 kDa
50 kDa
peIF2a
eIF2a
Tubulin

Figure S7

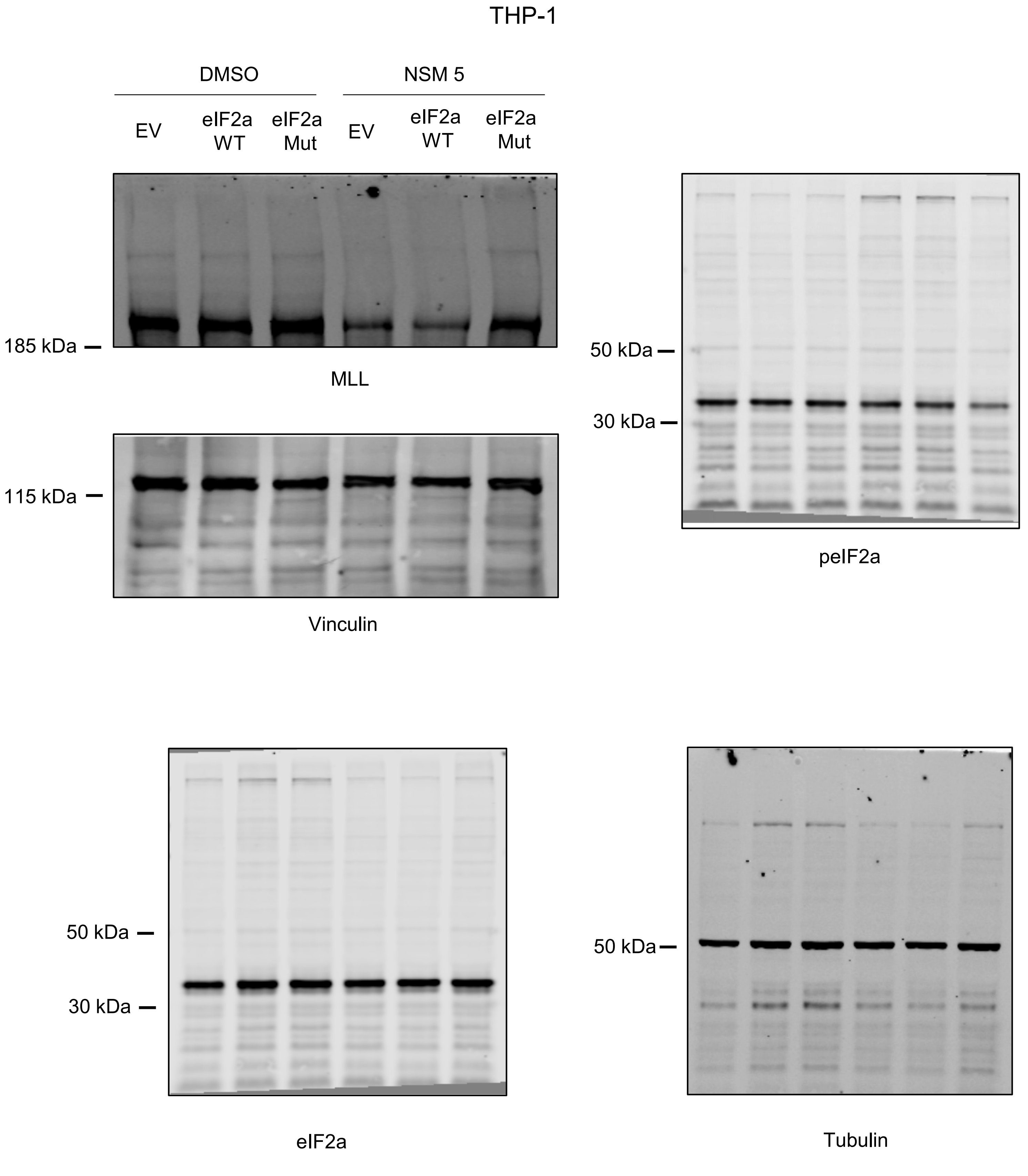
THP-1
DMSO
NSM 5
EV
eIF2a WT
eIF2a Mut
EV
eIF2a WT
eIF2a Mut
185 kDa
MLL
115 kDa
Vinculin
50 kDa
30 kDa
peIF2a
50 kDa
30 kDa
eIF2a
50 kDa
Tubulin

Figure S8

Supplementary Table S1. Genesets used in this study

Overlap of MLL-AF9 target genes (Prange et al, 2017) and genes downregulated by inhibition of MLL-AF9 (Zuber et al, 2011)	Overlap of MLL-AF9 target genes (Prange et al, 2017) and genes downregulated by inhibition of MLL-ENL & MLL-AF9 (Walf-Vorderwuelbecke, 2018)
ACAT2	ACAT2
AKAP1	AKAP1
ASH2L	CDK6
ATP1B1	DUSP3
BCL2	EEF1G
CALU	EXOSC3
CDCA5	GTF3A
CDK6	HOXA9
DCPS	IQCG
DECR1	IRAK1BP1
E2F7	KCNQ5
EEFSEC	MATN2
EIF3E	MRPL3
EIF4EBP1	MRPL9
ELF1	MYB
FADS2	MYC
FAF1	NT5DC2
FBXO5	NUCKS1
FOXD1	PARP1
GFI1	PCYOX1L
GGA2	PES1
GTF3A	PPIF
H2AFY	PPM1F
HOXA9	PVT1
INTS7	RCL1
MEX3A	SASH1
MYB	SLC16A1
MYC	SLC29A1
NT5DC2	SLC7A6
NUCKS1	STT3A
NUDT3	TMEM206
OAF	TOP1MT
PARP1	UCK2
PBX3	WDR77
PCYOX1L	ZEB2
PECAM1	
PPIF	
PRDX3	
PSMB4	
QSOX2	
RAD54L	
RCL1	
RPA3	
RPS9	
SCCPDH	
SIGMAR1	
SLC16A1	
SLC38A1	
SLC7A6	
SQLE	
SYNGR2	
TAGLN2	
TOP1MT	
UBAC1	
UBAP2	
UCK2	
